# Editorial Note: Fitting Membrane Resistance along with Action Potential Shape in Cardiac Myocytes Improves Convergence: Application of a Multi-Objective Parallel Genetic Algorithm

**DOI:** 10.1371/journal.pone.0322603

**Published:** 2025-04-09

**Authors:** The PLOS One Editors

The first three paragraphs of the Introduction section of this article [1] reuse text from a previously published article [2], which is cited as reference 14 in [1]. Specifically, sentences 1–2 of paragraph 1, sentences 3–5 of paragraph 2, and the entire third paragraph of Introduction are similar to or overlap verbatim with text from [2].

Additionally, the placement of the in-text citation to reference 14 in paragraph 3, sentence 2, of the Introduction incorrectly suggests that the limitation described in the third sentence of the paragraph applies to the work reported in [2] (reference 14 of [1]).

The *PLOS One* Editors issue this Editorial Note to inform readers of the above issues.
